# The Senile Heart

**Published:** 1894-11-03

**Authors:** 


### The Hospital

An Institutional Journal of

The Medical Sciences and Hospital Administration, with

AN EXTRA NURSING SUPPLEMENT.

Vol. XVII.—No. 423.] [Nov. 3, 1894.

### The Senile Heart.

Dr. G. W. Balfour has just given to the profession a little book on the "Senile Heart."

The author is himself an aged man, for it wants but a few months of half a century since he took his M.D. degree. His experience, both subjective and objective, has therefore been competent and long. One of the very first things Dr. Balfour tells us is that, of the uncounted millions who are born into the world, a mere fraction die of old age. The overwhelming majority succumb to disease in one or other of its protean forms. Nevertheless, he is fully convinced that if humanity could have the guidance of true science from the cradle to the grave this condition of things would be exactly reversed. The few it would then be who would die of disease; the vast majority would survive to old age. The Registrar-General tells us that "of 100,000 born in this country, 25,000 die before they have reached their fifth year, and 50,000 before they have reached their fiftieth. As many as 1,100 will survive to the age of ninety, but only two, like the last barques of an innumerable convoy, will reach the advanced age of a hundred and five." Science, if the whole population will but go to school to her, will make a very resolute effort to put another face upon all this.

There are two parts of the human organism, Dr. Balfour tells us, which if wisely used "largely escape senile failure." These two are the brain and the heart. Persons who think have often wondered why brain workers, great statesmen and others, should continue to work with almost unimpaired mental activity and energy up to a period when most of the organs and functions of the body are in a condition of advanced senile decay. There is a physiological reason for this, and Dr. Balfour tells us what it is. The normal brain, he affirms, "remains vigorous to the last," and that "because its nutrition is specially provided for." About middle life or a little later the general arteries of the body begin to lose their elasticity and to slowly but surely dilate. They become, therefore, much less efficient carriers of the nutrient blood to the capillary areas. But this is not the case with the internal carotids, which supply the capillary areas of the brain. On the contrary, those large vessels "continue to retain their pristine elasticity, so that the blood pressure remains normally higher than within the capillary area of any other organ in the body. The cerebral blood paths being thus kept open, the brain tissue is kept better nourished than the other tissues of the body." Who is there among those who have reached or passed middle age that will not be rejoiced to find such admirable physiological warrant for the belief that the brain may continue to work and even to improve almost to the very last hour of life?

What the brain is to the mind the heart is to the bodily organism. The heart, as is well known, tends to enlarge with advancing age, and its walls to get thicker. This hypertrophy has been very generally held to be pathological. Not so, contends Dr. Balfour. Such hypertrophy is practically universal among people in advancing years; and if it be universal it is manifest that it is developmental, and therefore normal. Dr. Balfour is aware that he is advancing views somewhat contrary to those generally accepted; and he therefore fortifies the conclusions of his own very wide experience by quoting those held by such eminent cardiologists as Bizot, Charcot, Cohnheim, and others; and it must be admitted that he makes out an excellent case for his most cheering and encouraging doctrine.

As in the case of the brain, there seems to be excellent physiological warrant for the conclusion that *caeteris paribus* the aged heart succeeds to, at any rate, a relative increase of strength as time goes on. The physiology is simple and obvious. In age the arteries are less elastic than in youth and middle life, and the blood pressure in them rises. This throws increased work upon the heart, and the increased effort, which Dr. Balfour assures us the organ is well able to make, since in the normal condition it always works well within its resources, results in a hypertophy which makes it relatively stronger than it has ever been before. One immediate effect of this is the more energetic flushing of the myocardium, or heart muscle itself, with an abundant supply of well-oxydised blood, and its consequent improved nutrition. Moreover, the total hæmoglobin of the blood, according to Leichtenstern, is absolutely increased after the age of sixty, and in these two factors we have all the necessary conditions of a permanently improved cardiac well-being. From these and from other considerations Dr. Balfour deduces the comfortable conclusion that old age in healthy persons should be a bright and cheerful period, a period of sound health in heart and brain, and of great competence for intllectual work.

The cheerful side of the picture has been purposely presented. But there is another side, and a side which is most melancholy reading. The reverse of the picture presents to us the abnormal and unhealthy senile heart, a heart bankrupt by heredity, disease, or foolish or wicked living. That side it is not necessary to enlarge upon for medical readers. But just as science teaches us the nature and capabilities of the normal heart, so she makes clear to the observing mind the practical difficulties of the abnormal organ, and also the rational methods of meeting and minimising them. What those difficulties are and what the means of their amelioration Dr. Balfour has expounded at length. The net result of his investigations is to give increased confidence to medical men in the resources of their art, and greater courage and hope to all sorts and conditions of aged persons, whether their hearts be sound or unsound.

